# Retrospective analyses of optical coherence tomography in recurrent macular edema following intravitreal therapy in patients with retinal vein occlusion

**DOI:** 10.1186/s12886-015-0107-y

**Published:** 2015-09-04

**Authors:** Stephen M. Holland, David G. Dodwell, Darrel A. Krimmel, Christopher M. de Fiebre

**Affiliations:** Illinois Retina Center, Springfield, IL 62704 USA; Southern Illinois University School of Medicine, Springfield, IL 62702 USA; Department of Ophthalmology, Loyola University Stritch School of Medicine, 2160 S First Ave, Maywood, IL 60153 USA; Krimmel Consulting, 98 Laconwood Dr., Springfield, IL 62712 USA; CMdeF Consulting, PO Box 112635, Upper St. Clair, PA 15241 USA

## Abstract

**Background:**

Optical coherence tomography has focused mainly on central subfield thickness to quantify macular edema in central and branch retinal vein occlusion. We examined macular fields other than the central subfield to determine if they are possibly independent indicators of recurrent macular edema.

**Methods:**

Single center, retrospective, consecutive case study of patients with recurrent macular edema secondary to either central or branch retinal vein occlusion. Thickness estimates of serial domain optical coherence tomography macular fields were obtained at the time of recurrent macular edema and analyzed retrospectively. Changes were expressed as a percentage of previous baseline levels. Change in thickness at each retreatment episode as well as average changes in thickness were calculated for each macular field for each eye. Data were analyzed via analysis of variance and Fisher’s post hoc analyses. The macular field which most frequently had the largest percent increase at the time of recurrence was also assessed using averages for each subject as well as for each retreatment episode. Individual episodes of recurrent macular edema were also examined to ascertain the frequency in which there was minimal foveal edema (<15 μm increase), but non-foveal edema was considered severe enough to warrant retreatment.

**Results:**

429 episodes of recurrent macular edema in 80 eyes were examined. In addition to the central subfield, the average mean change in thickness of the most affected quadrant (central vein occlusion) or hemisphere (branch vein occlusion) of the extrafoveal 3 mm band had the largest mean changes and also most frequently had the largest increases at the time of recurrent macular edema. In approximately 20 % of both central and branch occlusions, recurrent macular edema was detected in non-central macular fields in the absence of significant edema in the central subfield.

**Conclusions:**

Analyses of non-central macular fields as well as the central subfield may be useful in the early detection and treatment of recurrent macular edema in retinal vein occlusion.

## Background

Retinal vein occlusion (RVO) is one of the most prevalent retinal vascular disorders [1]. RVOs are classified based upon the anatomical location of the thrombus as either a central (CRVO) or a branch retinal vein occlusion (BRVO). Macular edema (ME) secondary to RVO results in part from capillary endothelial damage and breakdown of the blood retinal barrier [2]. The identification and quantification of the degree of ME has been greatly enhanced with the introduction and widespread use of optical coherence tomography (OCT) [3].

Large-scale clinical trials have demonstrated the utility of intravitreal injections of anti-vascular endothelial growth factor (anti-VEGF) agents or corticosteroids in treating ME associated with RVO [4–10]. These studies as well as clinical trials in other vitreoretinal diseases have almost exclusively utilized OCT to focus on measures of central subfield (i.e., foveal) thickness (CST), or related measures of foveal thickness. CST, however, is only one of many macular fields which can be analyzed with OCT. We do not know if these other macular fields may be sensitive indicators of recurrent macular edema (RME) in CRVO and BRVO.

The BRAVO [11] and CRUISE [12] studies of ranibizumab, the COPERNICUS [13] study of aflibercept and the GENEVA [14, 15] study of the intravitreal dexamethasone implant have all suggested that earlier treatment of ME secondary to RVO may provide improved efficacy of therapy. It has been hypothesized that the longer duration of ME associated with delayed treatment in sham treated patients in these studies resulted in irreversible damage to the retina which decreased the degree to which vision could be restored with pharmacotherapy [11, 13]. We hypothesize that the cumulative effect of multiple episodes of RME also may lead to damage which negatively affects final visual acuity outcomes, but that damage might be minimized with early detection and treatment of RME. An assessment of whether measurements in non-CST fields might be useful in the timely identification of RME has not been investigated and was a goal of this study.

The Zeiss Cirrus Model 4000 spectral domain high definition OCT (SDOCT) utilized in this study was able to compile 65,000 independent data points to create a topographical map of the macula. In addition to CST, thickness estimates were generated for multiple other fields encompassing different areas of the macula. The purpose of this study was to assess changes in thickness of individual macular fields at the time of RME and to determine if non-CST macular fields might also be sensitive indicators for RME following intravitreal therapy in patients with CRVO and BRVO.

## Methods

The study was a single center, retrospective, consecutive case study of 429 episodes of RME in 80 eyes of 79 patients with diagnosed ME secondary to either CRVO or BRVO. The study was approved by the Springfield Committee for Research Involving Human Subjects at Southern Illinois University School of Medicine. Eyes from patients with exudative age-related macular degeneration or advanced diabetic retinopathy were excluded. Eyes were also excluded if extensive previous laser resulted in severe macular scarring. Patients treated with panretinal photocoagulation to ischemic peripheral retina or limited focal laser to individual microaneurysms or in a light grid pattern were not excluded. Eyes at the time of inclusion in the study had previously been treated in our center with one or more intravitreal therapies including: dexamethasone intravitreal implant (Ozurdex®, Allergan, Irvine, CA USA), triamcinolone acetonide (2–8 mg; Kenalog®–40, Bristol-Myers Squibb, Princeton, NJ USA; Leiter’s Compounding Pharmacy, San Jose, CA USA), bevacizumab (1.25 mg; Avastin®, Genentech, South San Francisco, CA USA), ranibizumab (0.5 mg; Lucentis®, Genentech), aflibercept (2 mg; Eylea®, Regeneron, Tarrytown, NY USA) or a combination of intravitreal agents. If a patient was treated with a combination of intravitreal agents, each agent was administered in a separate treatment session. The decision to treat was made by the treating physician at the time of detection of disease recurrence. OCT scans were performed on a Zeiss Cirrus™ Model 4000 spectral domain HD-OCT (Dublin, CA USA). Patients returned for follow-up evaluation every 3–5 weeks after treatment.

Multiple OCT-derived values were generated for each scan with values corresponding to the average thickness of a macular field. The CST corresponds to the 1 mm diameter center of the fovea and is surrounded by concentric bands of 3 mm and 6 mm. In CRVO patients, each of these concentric bands was further divided into four quadrants: superior, inferior, nasal, and temporal where average thickness values were generated. For a given retreatment episode, the 3 mm quadrant having the greatest percent increase was defined as 3Q and the 6 mm quadrant displaying the greatest percent increase was defined as 6Q. In BRVO, it has been reported that ME is usually restricted to the superior or inferior hemispheres [16]. Hence, in BRVO, the 3 mm and 6 mm hemispheres with the most active disease were defined as 3H and 6H, respectively. Values for cube volume (CV) and cube average thickness (CAT) were also generated.

Following each intravitreal treatment for ME secondary to RVO, SDOCT topographical and raster line scan images obtained during follow-up visits were analyzed retrospectively to assess the date of greatest decrease in thickness prior to the next episode of RME. OCT data on the date of greatest reduction of ME served as new baselines for comparison with SDOCT data subsequently collected at the time of the next episode of RME. For the purpose of our study, RME was defined as a recurrence of intraretinal or subretinal fluid on raster line-scan analysis which in the opinion of the treating physician required additional intravitreal therapy.

In order to provide relative levels of ME across all OCT-derived macular fields and across all treatments, SDOCT changes were expressed as a percentage of the most recently defined baseline levels. In addition to analyzing data for each episode of RME, an average change in thickness was calculated for each macular field for each eye to control for the different numbers of instances of RME for each eye. Data were compared via analysis of variance (ANOVA) followed by Fisher’s protected least squares difference (PLSD) *post hoc* analyses. In order to assess which macular fields most frequently had the largest increase at the time of RME, the field with the largest increase following each episode of RME and the field with the largest average increase for each eye was identified and assigned a value of 1. Other macular fields were assigned a value of −1. These data also were subsequently analyzed via ANOVA.

To ascertain the frequency in which there was minimal foveal RME, but non-foveal RME was considered severe enough to warrant retreatment, individual episodes of RME were examined and the number of episodes where retreatment occurred when changes in CST were less than 15 μm was calculated.

## Results

### CRVO

Analyses were performed on 213 episodes of RME and retreatment of 35 eyes of 35 patients with CRVO. The number of instances of RME per eye with CRVO ranged from 1–19 episodes (median = 6 episodes).

**Fig. 1:**
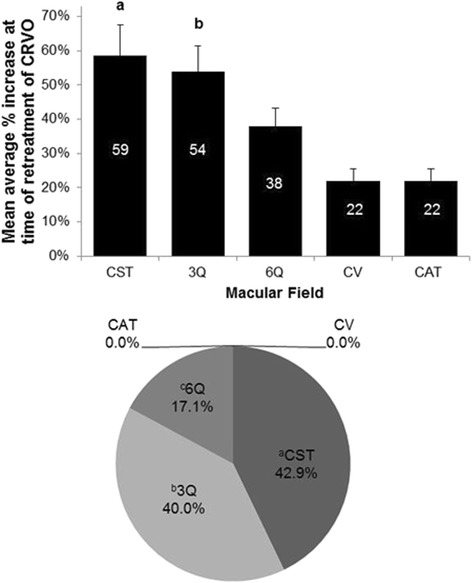
*Top panel*: Mean average percent change of OCT measurements of different macular fields at the time of retreatment for CRVO. An average percent change for each macular field was calculated for each subject for each macular field. Data are mean of these averages ± S.E.M. Percent values are displayed within each bar. ANOVA: main effect of macular field (F_4,170_ = 4.175; *P* < 0.0001). Fisher’s PLSD *post hoc* tests: ^**a**^ **CST** > 6Q, *P* = 0.019; (CV, CAT), *P* < 0.0001; ^**b**^ **3Q** > (CV, CAT), *P =* 0.0003. *Bottom panel*: Frequency that the average OCT values for different macular fields for each subject have the greatest percentage increase in patients treated for CRVO. ANOVA: main effect of macular field (F_4,170_ = 24.229; *P* < 0.0001). Fisher’s PLSD *post hoc* tests: ^**a**^ **CST** > 6Q, *P* = 0.0032; > (CV, CAT), *P* < 0.0001; ^**b**^ **3Q** > 6Q, *P* = 0.0085; > (CV, CAT), *P* < 0.0001; ^**c**^ **6Q** > (CV, CAT), *P* = 0.0475

Data where the average for each eye for each macular field were analyzed are shown in Fig. 1. As shown in the bar graph (top), CST and 3Q had significantly larger average increases in thickness than the other macular fields at the time of CRVO-associated RME with average mean increases of 58.6 and 54.0 % for CST and 3Q, respectively. 6Q had larger average increases than either CV or CAT.

Data were analyzed to ascertain which OCT derived value most frequently had the largest average increase at the time of RME and retreatment for CRVO (Fig. 1, lower panel). CST (42.9 %) and 3Q (40.0 %) most frequently had the largest average increases of any OCT derived value. 6Q had the largest average increase in 17.1 % of eyes.

**Fig. 2:**
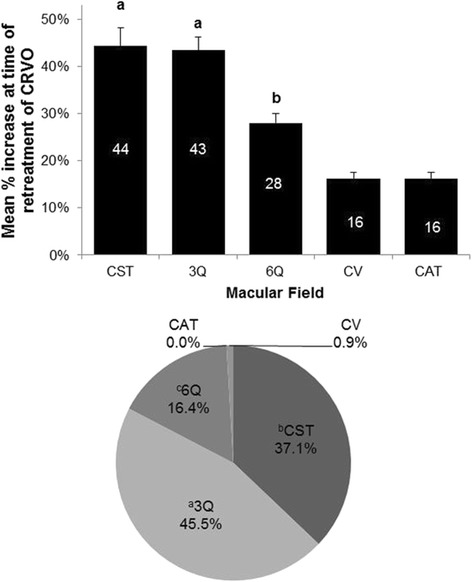
*Top panel*: Mean percent change of OCT measurements of different macular fields at the time of retreatment for CRVO. A percent change in OCT measurements at the time of retreatment was calculated for each macular field for each episode of recurrent macular edema. Data represent the mean ± S.E.M. Percent values are displayed within each bar. ANOVA: main effect of macular field (F_4,1060_ = 31.158; *P* < 0.0001). Fisher’s PLSD *post hoc* tests: ^**a**^ **CST = 3Q** > (6Q, CV, CAT), *P* < 0.0001; ^**b**^ **6Q** > (CV, CAT), *P* = 0.0008. *Bottom panel*: Frequency that OCT values for different macular fields have the greatest percentage increase at the time of retreatment for CRVO. ANOVA: main effect of macular field (F_4,1060_ = 72.600; *P* < 0.0001). Fisher’s PLSD *post hoc* tests: ^**a**^ **3Q** > CST, *P* = 0.0142; (6Q, CV, CAT), *P* < 0.0001; ^**b**^ **CST** > (6Q, CV, CAT), *P* < 0.0001; ^**c**^ **6Q** > (CV, CAT), *P* < 0.0001

OCT data for every CRVO-associated episode of RME for each macular field were also analyzed and are shown in Fig. 2. In the bar graph (top), CST and 3Q had significantly larger increases in thickness than the other macular fields at the time of RME with mean increases of 44.3 and 43.4 % for CST and 3Q, respectively. 6Q had larger increases than either CV or CAT.

Individual data were also analyzed to ascertain which OCT derived value most frequently had the largest increase at the time of RME and retreatment for CRVO (Fig. 2, lower panel). 3Q (45.5 %) most frequently had the largest increase in thickness at the time of RME. CST and 6Q had the largest increase in 37.1 and 16.4 % episodes of RME, respectively.

In CRVO, 23.0 % (49/213) of individual episodes of RME were accompanied by increases in CST of less than 15 μm. Most frequently, RME in 3Q and/or 6Q was seen warranting retreatment (data not shown).

### BRVO

Analyses were performed on 216 episodes of RME in 45 eyes of 44 patients with BRVO. The number of instances of RME per eye with BRVO ranged from 1–11 episodes (median = 4).

**Fig. 3:**
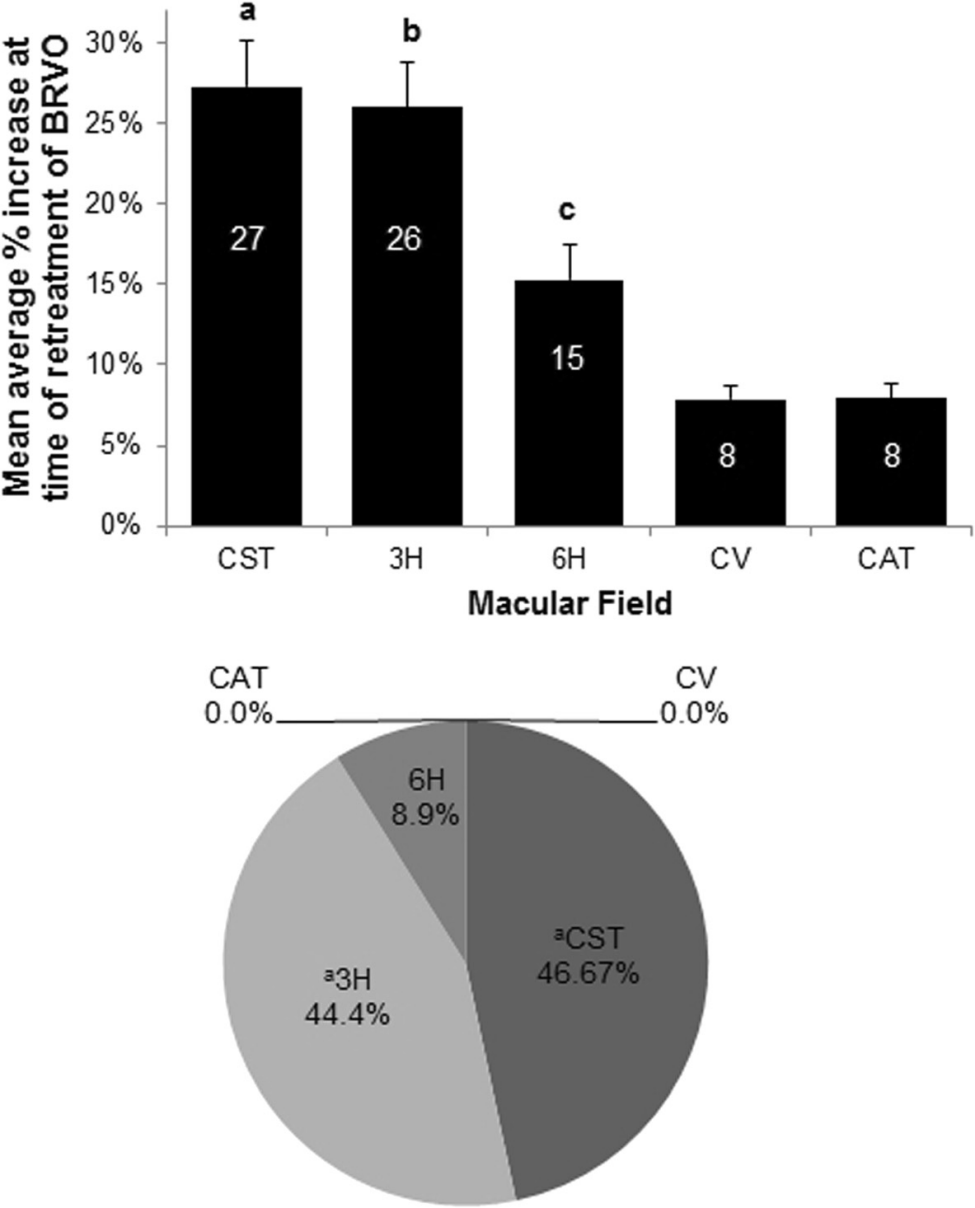
*Top panel*: Mean average percent change of OCT measurements of different macular fields at the time of retreatment for BRVO. An average percent change for each macular field was calculated for each subject for each macular field. Data are mean of these averages ± S.E.M. Percent values are displayed within each bar. ANOVA: main effect of macular field (F_4,220_ = 1.595; *P* < 0.0001). Fisher’s PLSD *post hoc* tests: ^**a**^ **CST** > (6H, CV, CAT), *P* < 0.0001; ^**b**^ **3H** > 6H, *P* = 0.0005; (CV, CAT), *P* < 0.0001; ^**c**^ **6H** > CV, *P* = 0.0143; CAT, *P* = 0.0157. *Bottom panel*: Frequency that the average OCT values for different macular fields for each subject have the greatest percentage increase in patients treated for BRVO. ANOVA: main effect of macular field (F_4,220_ = 40.178; *P* < 0.0001). Fisher’s PLSD *post hoc* tests: ^**a**^ **(CST = 3H)** > all other fields, *P* < 0.0001

Data where the averages for each eye for each macular field were analyzed are shown in Fig. 3. As shown in the bar graph (top), CST and 3H had significantly larger average increases in thickness than the other macular fields at the time of BRVO-associated RME with average mean increases of 27.3 and 26.0 % for CST and 3H, respectively. 6H had larger average increases than either CV or CAT.

Data were analyzed to ascertain which OCT derived value most frequently had the largest average increase at the time of RME and retreatment for BRVO (Fig. 3, lower panel). CST (46.7 %) and 3H (44.4 %) most frequently had the largest average increases of any OCT derived value. 6Q had the largest average increase in 8.9 % of eyes.

**Fig. 4:**
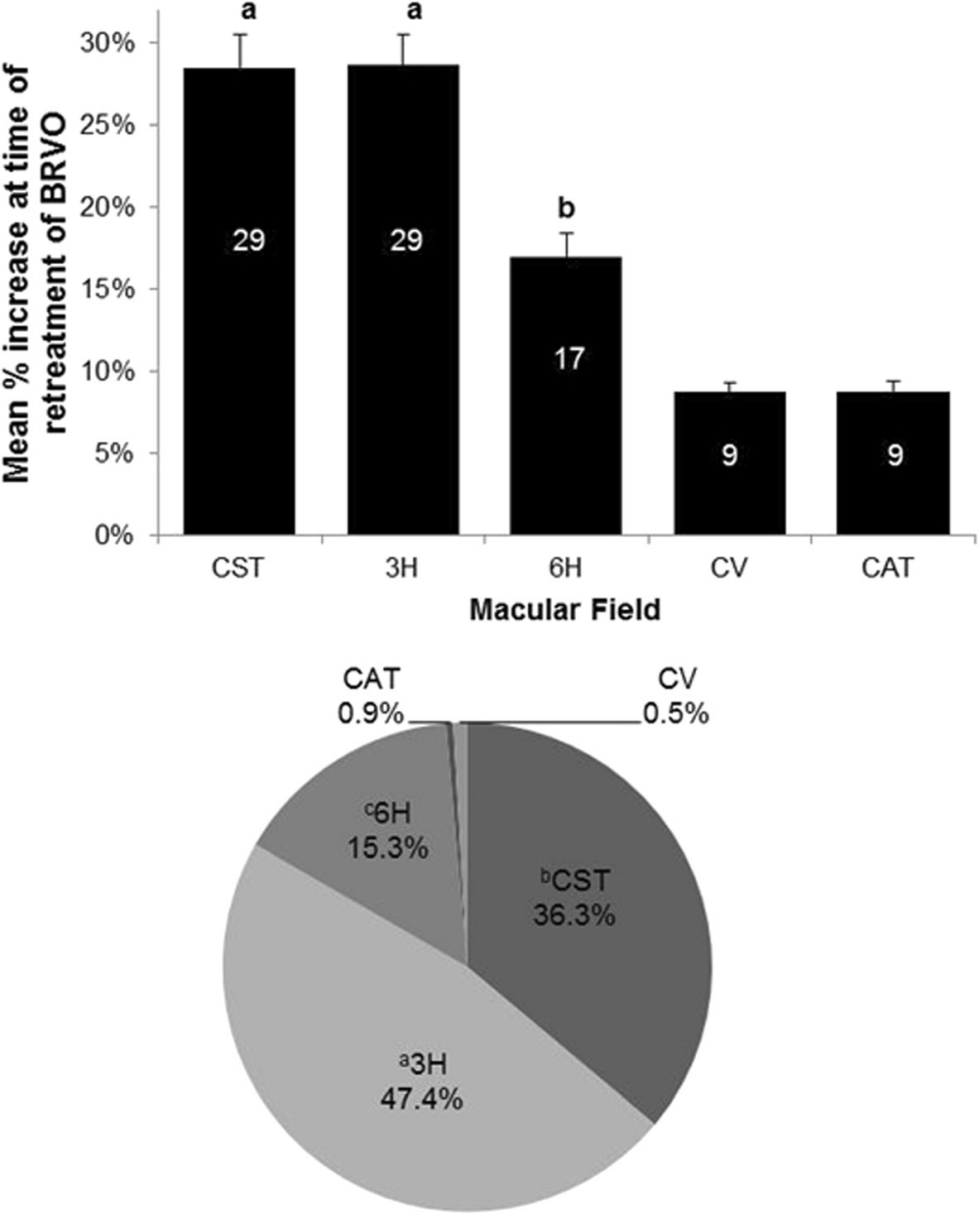
*Top panel*: Mean percent change of OCT measurements of different macular fields at the time of retreatment for BRVO. A percent change in OCT measurements at the time of retreatment was calculated for each macular field for each episode of recurrent macular edema. Data represent the mean ± S.E.M. Percent values are displayed within each bar. ANOVA: main effect of macular field (F_4,1066_ = 46.951; *P* < 0.0001). Fisher’s PLSD *post hoc* tests: ^**a**^ **CST = 3H** > (6H, CV, CAT), *P* < 0.0001; ^**b**^ **6H** > (CV, CAT), *P* < 0.0001. *Bottom panel*: Frequency that OCT values for different macular fields have the greatest percentage increase at the time of retreatment for BRVO. ANOVA: main effect of macular field (F_4,1070_ = 76.481; *P* < 0.0001). Fisher’s PLSD *post hoc* tests: ^**a**^ **3H** > CST, *P* = 0.0011; (6H, CV, CAT), *P* < 0.0001; ^**b**^ **CST** > (6H, CV, CAT), *P* < 0.0001; ^**c**^ **6H** > (CV, CAT), *P* < 0.0001

OCT data for every episode of BRVO-associated RME for each macular field were also analyzed and are shown in Fig. 4. In the bar graph (top), 3H and CST had significantly larger increases in thickness than the other macular fields at the time of RME with mean increases of 47.4 and 36.3 % for 3H and CST, respectively. 6H had larger average increases than either CV or CAT.

Individual data were also analyzed to ascertain which macular field most frequently had the largest increase at the time of RME and retreatment for BRVO (Fig. 2, lower panel). 3H (47.4 %) most frequently had the largest increase in thickness at the time of RME. CST and 6H had the largest increase in 36.3 and 15.3 % of episodes of RME, respectively.

In BRVO, 19.4 % (42/216) of individual episodes of RME were accompanied by increases in CST of less than 15 μm. Most frequently, RME in 3H and, to a lesser extent in 6H, were seen warranting retreatment (data not shown).

**Fig. 5:**
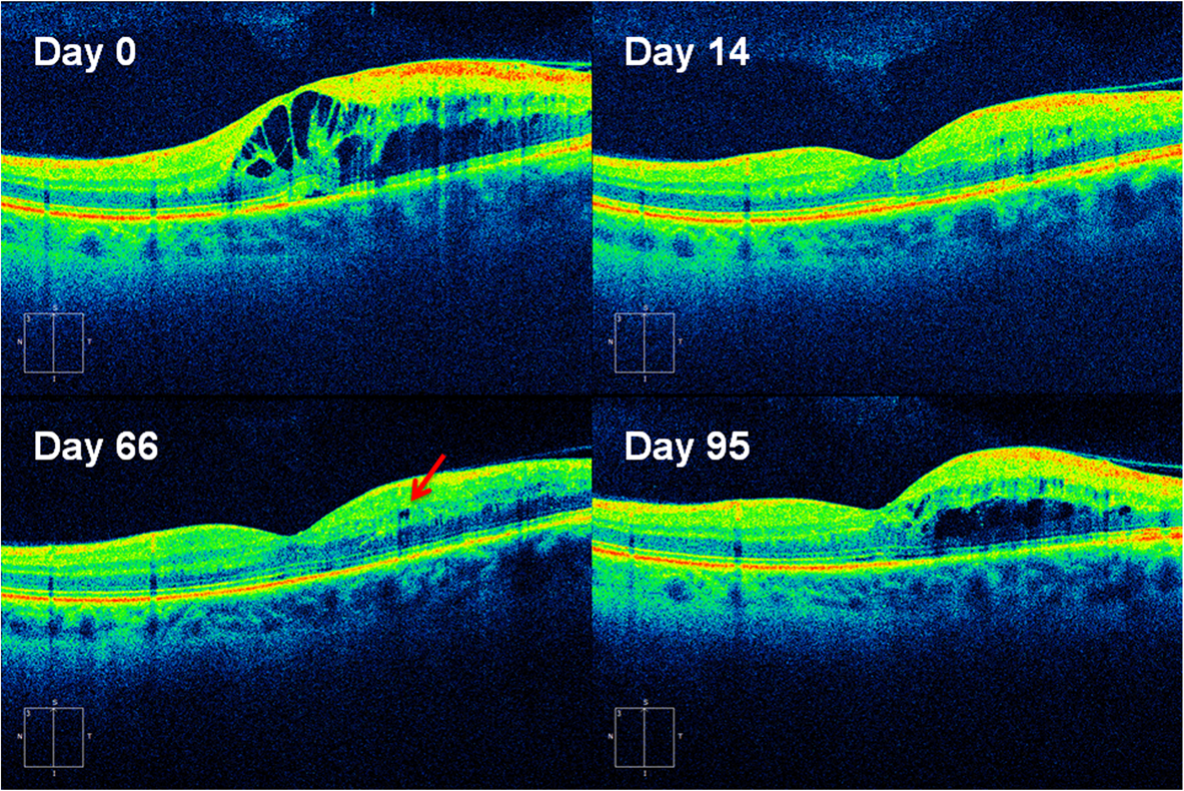
SDOCT scans from a patient with BRVO demonstrating intra-retinal fluid involving CST and the superior 3H field. The patient was diagnosed with BRVO and treated with the intravitreal dexamethasone implant (Ozurdex®) on Day 0 (*upper left*). On Day14 (*upper right*), ME had resolved. On Day 66 (*lower left*), fluid was increasing with a cyst noted in the superior 3H region (arrow), without any change in CST. Treatment was recommended, but the patient declined and elected to return in 4 weeks. On Day 95 (*lower right*), a 24.7 % increase in fluid was noted in 3H compared to a 14.6 % increase in CST

In Fig. 5, OCT scans are shown from a patient with BRVO in whom maximal increases developed in 3H at the time of RME. As shown in the upper right of the figure, ME had resolved within 14 days after treatment with the intravitreal dexamethasone implant. Approximately 9.5 weeks after treatment (lower left), fluid was noted to be increasing with a cyst observed in the 3H field. Retreatment was recommended, but the patient elected observation with follow-up in 4 weeks. On Day 95 post-treatment, pronounced RME was seen with a 24.7 % increase in 3H (479 μm at treatment compared to a baseline of 384 μm). Increased foveal edema was also present as shown by a 14.6 % increase in CST (337 μm at treatment compared to a baseline of 294 μm).

## Discussion

Few studies have examined non-foveal OCT fields as indicators of RME in CRVO and BRVO. In this study, ME was analyzed in the 1.5 mm CST region most closely associated with the fovea as well as in the 3 and 6 mm parafoveal and perifoveal regions. The data presented here demonstrate that in addition to CST, the largest increases in RME occurred in 3Q in CRVO and 3H in BRVO. To a lesser extent, 6Q and 6H showed sizable increases. These data suggest that non-CST fields might be predictive of early RME in CRVO and BRVO.

In both CRVO and BRVO, the average percent increases for CST and 3Q/3H, the fields with the largest average increases in RME, were not statistically different from each other if every episode of RME was included in analyses as well as if an average for each field for each eye was used for analyses. Further, probabilities that they differed from each other were very high (*P* > 0.80) rendering the possibility of a type ii error very unlikely. When average changes for each field in each eye were used for analyses, the frequency (% of subjects) for which 3Q in CRVO and 3H in BRVO had the largest average increase did not differ significantly from the frequency for which CST had the largest average increase. When each episode of RME was used in analyses, however, 3Q and 3H were significantly more frequently detected as having a greater percent increase in thickness than CST. This latter finding would also suggest that a type II error was not committed in rejecting that CST has greater percent increases in thickness compared to 3Q/H. Collectively, these data suggest that assessment of RME in the proximal, extrafoveal 3 mm band is at least equally sensitive as an indicator of RME as the fovea-associated CST field. The fields associated with the extrafoveal 6 mm band also had large average increases in some subjects and, in several subjects, these increases were greater than any other macular field. Taken as a whole, these data show that some subjects develop greater or earlier RME in one field versus another (including non-CST fields).

As noted, there were sizable differences in the number of episodes of RME among the subjects in this study. In order to evenly weight data among all subjects regardless of the number of episodes of RME, a single average change in OCT value was calculated for each macular field for each subject and these averaged values were used for comparison among subjects for the data presented in Figs. 1 and 3. Findings from these analyses were compared with analyses where every episode of RME was equally weighted (Figs. 2 and 4). All analyses indicated that the 3Q/3H fields are as sensitive as CST as indicators of RME.

While a larger study with all subjects having multiple instances of RME would be required to statistically assess intra-individual differences in RME development, some insight was gained through an informal examination of the individual occurrences of RME in the subjects studied here. First, some subjects routinely show maximal RME in one field while the field with maximal RME development was less predictable in others. Second, in approximately 20 % of episodes of RME, sufficient increases in thickness were seen in non-foveal regions to warrant retreatment even though increases in CST were less than 15 μm. These observations demonstrate that CST should not be the sole macular field examined in the detection of RME in RVO.

Data from those individuals with at least 5 episodes of RME were also informally examined to assess if baseline values were increasing with each subsequent episode of RME. As opposed to a saw-tooth effect of progressively increasing macular thickness, most patients showed either a decreasing trend in macular thickness or a tendency to reach a relatively consistent baseline following each treatment.

Clinical trials have suggested that a delay in the treatment of ME in RVO leads to decreased efficacy [11–15]. Additionally, Scott et al. [17] reported in the SCORE study that longer duration of macular edema was a predictor of poorer CST outcomes. If use of multiple SDOCT macular fields leads to earlier detection and treatment of RME therapeutic outcomes may be improved.

Arema et al. [18] used OCT to compare the location of ME and vascular leakage in patients with BRVO and showed that RME tends to shift from extrafoveal fields toward the fovea with time. Early detection and retreatment of RME may prevent damage to central vision by arresting ME before it encroaches on the fovea. Although our study was not designed to evaluate this, the images shown in Fig. 5 support this in that a small amount of fluid was seen in the 3H region of a patient with BRVO that subsequently spread to involve the CST region. At the time of retreatment, the increase in fluid in 3H (24.7 %) was greater than the increase in CST (14.6 %).

Park et al. [19] examined extrafoveal changes following intravitreal bevacizumab in BRVO. The authors noted that bevacizumab affects the entire macula and that measurements beyond CST may be appropriate for monitoring therapeutic effects.

Noma et al. [20] conducted a multivariate analysis of data from patients with BRVO. Best corrected visual acuity was significantly correlated with thickness/volume in the CST, 3QS, 6QI and 3QN quadrants. In contrast, retinal sensitivity was correlated with thickness/volume in all macular fields examined. These data also demonstrate that RME is not limited to the CST.

In addition to the fovea, RVO-associated RME may damage the outer macula and the peripheral retina. Treatment of RME in the absence of foveal ME may help prevent loss of extrafoveal vision and prevent the spread of RME to the fovea.

Although patients included in this study were treated with a variety of intravitreal agents including anti-VEGF agents and steroids, the study was not designed to assess if RME following different treatments produced different patterns of RME development. A prospective study might be more suited to assess not only if different treatments produce differences in where RME subsequently develops, but also in assessing if there are anatomical differences in how ME regresses following treatment with these different agents.

While OCT provides quantitative measurements of retinal thickness, in display mode it also allows for the visual detection of small amounts of intra-retinal or sub-retinal fluid which may not be large enough to detect overall thickness increases within a macular field. As such, both quantitative and qualitative information provided by OCT can be used in determining the need for retreatment and either or both may show involvement of non-CST fields.

The principal limitations of this study are its retrospective nature, size, disparate numbers of episodes of RME among subjects and data being limited to a single practice/physician. It is uncertain if similar results would be obtained in a larger, multicenter, retrospective study given potential differences in physician treatment patterns. A multicenter, prospective study examining different OCT-based retreatment criteria could help to optimize the treatment of RME associated with RVO.

A strength of the study is the consistent scheduling of follow-up visits within the practice that included SDOCT at each visit to detect subtle RME. Also, each patient’s prior history regarding the duration of therapy and timing of RME was used to plan follow-up at time periods closest to those of anticipated disease recurrence (i.e., at time of RME).

## Conclusions

Of the many risk and prognostic factors associated with RVO, the duration of untreated ME may be most easily modified by the treating ophthalmologist. The data presented here demonstrate that an increase in fluid can occur outside of and in the absence of changes in CST. The examination of multiple macular fields, including CST, via SDOCT may allow for earlier detection of RME. Earlier detection could lead to more timely treatment and a possible decrease in the cumulative duration and damage from RME thereby improving long-term visual outcomes.

## Abbreviations

OCT: Optical coherence tomography
SDOCT: Spectral domain optical coherence tomography
CRVO: Central retinal vein occlusion
BRVO: Branch retinal vein occlusion
RVO: Retinal vein occlusion
ME: Macular edema
RME: Recurrent macular edema
anti-VEGF: anti-vascular endothelial growth factor
CST: Central subfield thickness
3Q: 3 mm quadrant having the greatest percent increase in thickness
6Q: 6 mm quadrant with the greatest percent increase in thickness
3H: 3 mm hemisphere having the greatest percent increase in thickness
6H: 6 mm hemisphere with the greatest percent increase in thickness
CV: Cube volume
CAT: Cube average thickness
ANOVA: Analysis of variance
